# Case Report: Favorable outcome in a patient with simultaneous dengue meningoencephalitis and Wernicke’s thiamine deficiency

**DOI:** 10.3389/fmed.2025.1515845

**Published:** 2025-06-17

**Authors:** Saket Satyasham Toshniwal, Jiwan S. Kinkar, Sourya Acharya, Sunil Kumar

**Affiliations:** ^1^Department of General Medicine, Jawaharlal Nehru Medical College, Datta Meghe Institute of Higher Education and Research, Wardha, India; ^2^Department of Neurology, Jawaharlal Nehru Medical College, Datta Meghe Institute of Higher Education and Research, Wardha, India

**Keywords:** dengue, thiamine, dengue meningoencephalitis, Wernicke’s encephalopathy, alcoholism, fever, thrombocytopenia

## Abstract

This case report describes the case of a 35-year-old man with a history of chronic binge drinking who presented with fever, associated confused state of mind, and symptoms indicative of acute alcohol withdrawal, including tremors and agitation. Routine investigations revealed pancytopenia and a positive dengue NS1 antigen test, confirming a diagnosis of dengue fever. Neurological examination revealed abnormalities, such as nystagmus, ophthalmoplegia, and altered mental status, which raised the initial suspicion of an alternative diagnosis of Wernicke’s encephalopathy (WE). Magnetic resonance imaging (MRI) revealed bilateral thalamic hyperintensities and meningeal enhancement, while the positive dengue status revealed a rare manifestation of dengue meningoencephalitis. However, owing to the patient’s history of chronic alcoholism, WE could not be completely ruled out. The diagnostic challenge in distinguishing between WE and dengue-associated neurological complications is highlighted by the overlapping symptoms in this case. Furthermore, early administration of thiamine played a crucial role in managing the patient’s condition, highlighting the importance of timely intervention in such complex cases marked by diagnostic uncertainties.

## Introduction

Two neurological conditions characterized by bilateral thalamic lesions are dengue encephalopathy (DE) and Wernicke’s encephalopathy (WE), whose etiologies are distinct but share similar clinical and radiological features. Dengue encephalopathy is an infrequent but serious complication of dengue fever, characterized by altered mental status, seizures, and other neurological deficits. In such cases—often showing bilateral thalamic involvement on MRI, reflecting viral invasion or immune-mediated brain injury—we frequently observe brain involvement accompanied by characteristic metabolic abnormality on PET. Patients with systemic dengue symptoms in dengue-endemic regions, particularly those presenting with encephalitis, are of special concern (1).

However, Wernicke’s encephalopathy is a metabolic disease caused by thiamine deficiency, which commonly affects individuals with chronic alcoholism. It presents with confusion, ocular disturbances, and ataxia, and typically shows symmetric hyperintensities in the thalamus (and other midline structures) on MRI. Differentiating between the two conditions is challenging, further complicated by the presence of overlapping risk factors such as alcoholism and dengue exposure; both conditions share the characteristic finding of bilateral thalamic lesions (2).

WE is a diagnosis of urgent importance, where prompt thiamine replacement is essential to prevent irreversible damage, whereas dengue encephalopathy require supportive care. Distinguishing between these two serious conditions depends on clinical history, laboratory results, and neuroimaging findings (2).

Most importantly, this case illustrates the difficulty in diagnosis and distinguishing WE from dengue-related encephalitis, as both share common neurological symptoms. It underscores the importance of considering early administration of thiamine despite diagnostic uncertainty. Increasing awareness among physicians to initiate early administration of thiamine in complex case scenarios to prevent further neurological deterioration is of the utmost importance, particularly in cases with overlapping encephalopathies with diagnostic uncertainties (1).

## Case presentation

This case report presents the case of a 35-year-old man with a decade-long history of chronic binge drinking, who was admitted with a fever of 102°F, in a confused state, and exhibited physical signs consistent with alcohol withdrawal, including tremors and agitation. His last alcoholic drink was 2 days prior to his admission. His Clinical Institute Withdrawal Assessment for Alcohol, revised (CIWA-Ar) score was calculated based on the clinical signs, and the score was 20, which suggested severe withdrawal. On physical examination, he appeared toxic—i.e., his overall appearance upon presentation indicated a critically ill clinical state based on the clinical signs and vitals, disoriented, and confused, with a high-grade fever, tachycardia, a blood pressure of 80/40 mm of Hg, and a respiratory rate of 22 cycles per minute.

On neurological examination, the patient was disoriented to time and place with positive signs of meningeal irritation. Motor examination revealed normal muscle strength, graded as 5/5 on the Medical Research Council scale, and brisk deep tendon reflexes in all four limbs with a positive Babinski’s sign. Sensory examination could not be assessed. However, nystagmus and an unsteady gait were observed with signs of ophthalmoplegia. At presentation, the patient exhibited bilateral horizontal gaze palsy, suggesting the involvement of supranuclear pathways rather than an isolated sixth nerve palsy. Vertical movements were relatively preserved.

His complete blood count showed low hemoglobin and platelets, alongside a reduced white blood cell count, suggesting pancytopenia. The differential count showed relative lymphocytosis (50%), neutropenia (40%), and mild monocytosis (6%), with normal eosinophils and basophils, consistent with a viral etiology and bone marrow suppression. The patient underwent testing for malaria (peripheral smear and rapid diagnostic test), leptospirosis (IgM serology), typhoid (Widal test and blood cultures), herpes simplex virus (HSV PCR), and tuberculosis (CSF GeneXpert)—all of which returned negative. Liver function tests displayed mildly elevated transaminases, with a normal renal function test and normal serum electrolytes. The serum thiamine level was 30 nmol/L (reference range: 70–180 nmol/L), measured using high-performance liquid chromatography (HPLC). The serum vitamin B12 level was 140 pg./mL (reference range: 200–900 pg./mL), assessed via chemiluminescent immunoassay (CLIA).

The patient tested positive for dengue NS1 antigen on day 3 of fever, which, together with fever and pancytopenia, aligned with a diagnosis of dengue with encephalopathy. However, owing to his chronic alcoholism history with severely deficient thiamine and B12 levels and positive neurological findings, a probable diagnosis of Wernicke’s encephalopathy was also considered.

**Figure 1:**
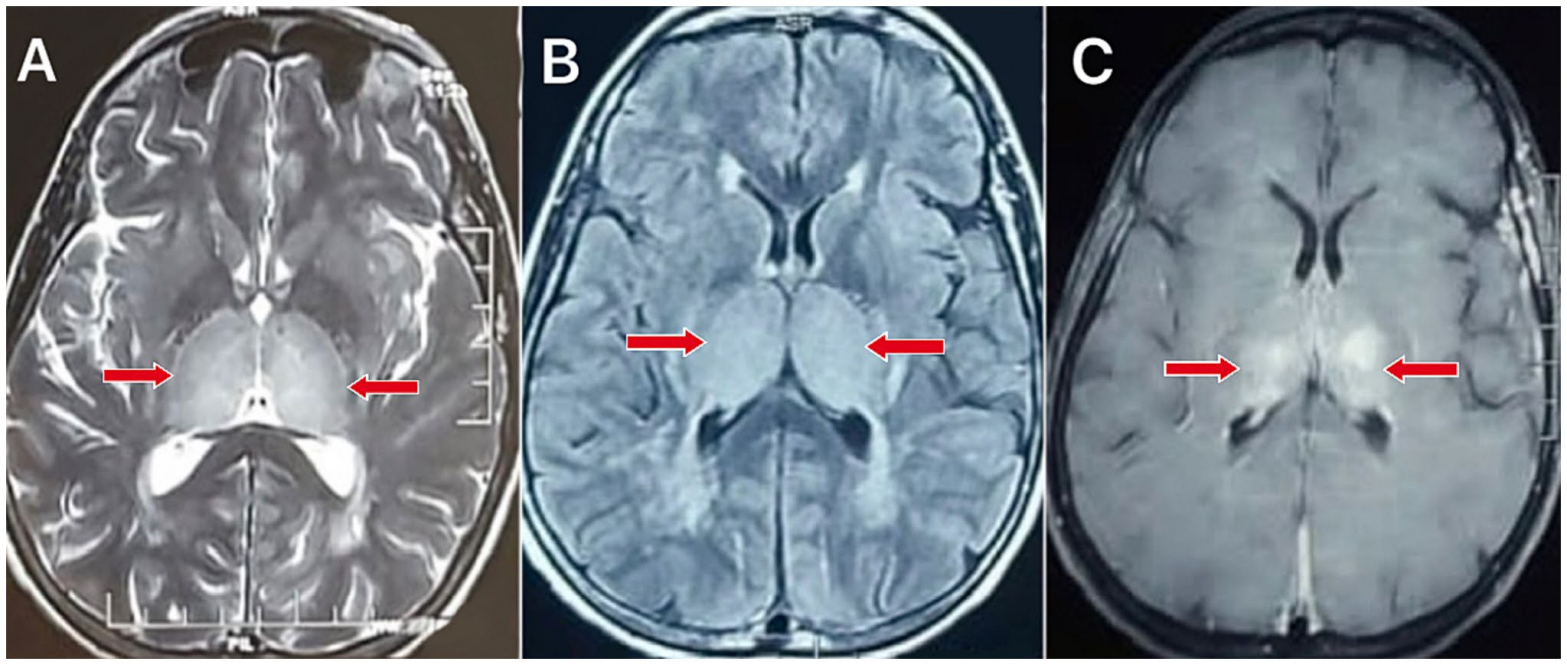
Axial MRI images of the brain demonstrate bilateral symmetrical lesions (indicated by red arrows) in the thalamus across different sequences: **(A)** T2-weighted image shows hyperintense lesions in the thalamus. **(B)** Fluid-Attenuated Inversion Recovery (FLAIR) sequence reveals prominent hyperintense lesions in the thalamus. **(C)** Diffusion-weighted imaging (DWI) sequence shows restricted diffusion in the thalamic lesions.

**Figure 2:**
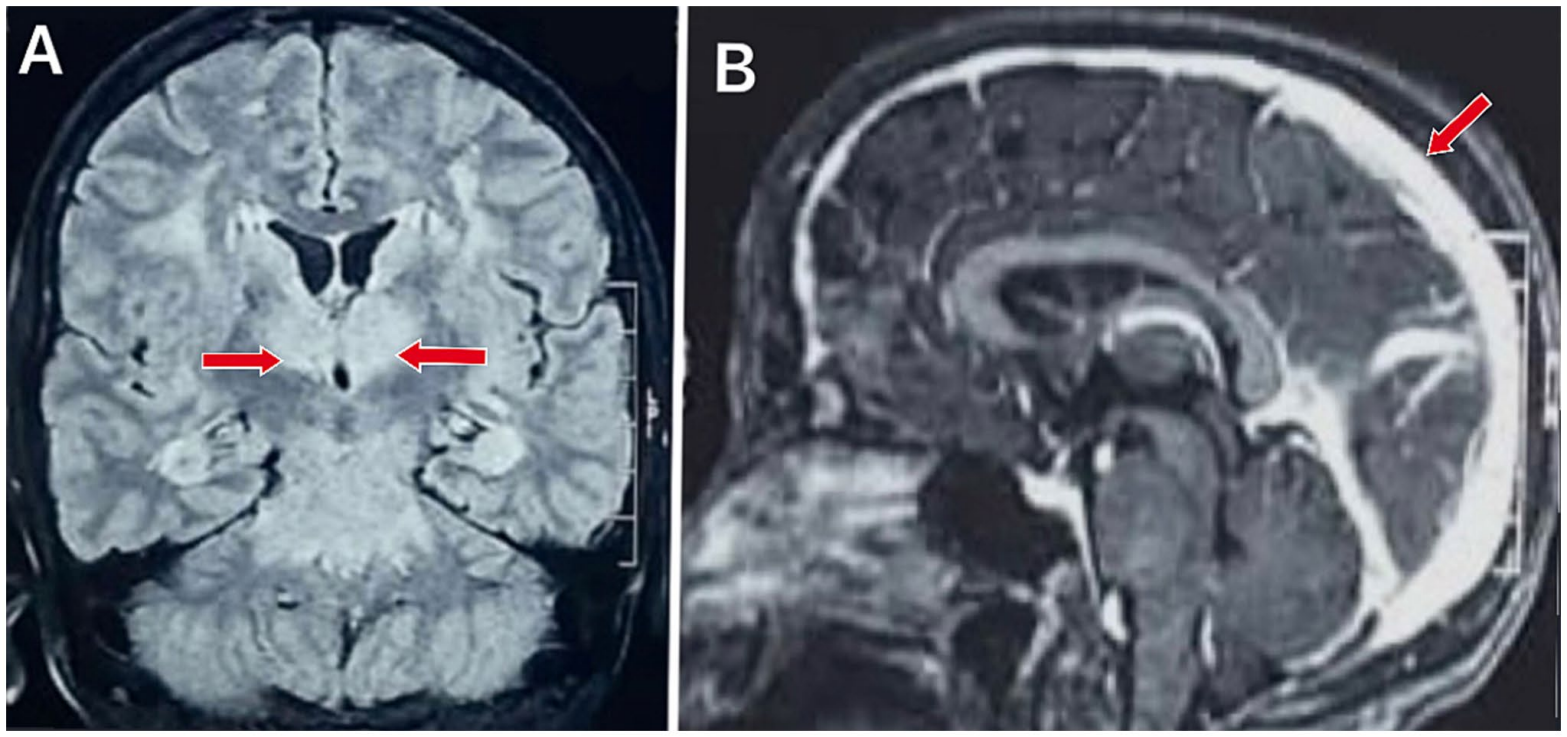
Coronal and sagittal MRI images of the brain show pathological findings: **(A)** Coronal Fluid-Attenuated Inversion Recovery (FLAIR) sequence demonstrates bilateral symmetrical hyperintense lesions in the thalamus (red arrows). **(B)** Sagittal post-contrast T1-weighted image shows contrast enhancement in the meninges (red arrow), suggesting meningeal involvement.

MRI brain screening revealed bilateral thalamic hyperintense lesions with meningeal enhancements, as shown and explained in Figures 1, 2.

All of these findings can be associated with both WE and dengue encephalitis. Given the lack of involvement of the mammillary body and systemic infection, dengue encephalitis was considered the main diagnosis. For the sake of the patient’s alcohol history, however, the diagnosis of WE could not be entirely ruled out. The diagnosis of suspected WE, in this case, was supported using Caine’s criteria, which require the presence of one risk factor (chronic alcoholism in this case) plus two clinical features of the classic triad (ophthalmoplegia, ataxia, and altered mental status), which were fulfilled in this case.

The cerebrospinal fluid analysis further showed moderately elevated cells (50 cells/μL) with moderately elevated protein (60 mg/dL) and normal glucose levels. The CSF was positive for dengue antigen by polymerase chain reaction, which helped reach the diagnosis of dengue meningoencephalitis.

While Wernicke’s encephalopathy (WE) typically does not present with fever or thrombocytopenia, making it an unlikely primary cause of these symptoms, the possibility of its co-existence with an infectious etiology, such as dengue, should be considered given the overlapping neurological manifestations.

Both conditions were treated in the patient due to the uncertainty of the diagnosis and the overlap of sysmptoms. In the early stages of management, supportive care for dengue fever, consisting of intravenous fluids to prevent shock and antipyretics to control the fever, dominated the thinking of management. Empirical intravenous thiamine supplementation was initiated due to high clinical suspicion of Wernicke’s encephalopathy (WE), as the patient fulfilled Caine’s criteria (a validated tool for diagnosing WE in non-alcoholic and alcoholic populations). Although WE was not formally confirmed, according to the recent guidelines’ recommendations, the patient was administered intravenous thiamine at a dose of 500 mg three times daily for 3 days, followed by 250 mg once daily for 5 additional days, via slow IV infusion, in line with standard protocols for suspected acute WE. Thiamine was continued throughout the hospital stay and later transitioned to oral supplementation.

In addition, the patient was treated with intravenous lorazepam, starting at 2 mg every 6 h (total 8 mg/day) to manage alcohol withdrawal symptoms, with dosage adjusted based on the CIWA-Ar score. As his symptoms improved, the lorazepam was tapered over 5 days—first by reducing to 1 mg every 6 h, then 1 mg every 8 h, and finally transitioned to oral lorazepam 1 mg twice daily, which was tapered and stopped by day 7 of hospitalization. The patient’s fever improved, and his neurological status improved as well during his stay in the intensive care unit. After thiamine replacement, as gaze restriction improved, horizontal nystagmus became prominent, particularly in lateral gaze. This transition aligns with the well-documented phenomenon in B1 deficiency, where initial gaze restriction improves, but nystagmus—often of a horizontal nature—becomes evident during recovery. He was discharged after approximately 2 weeks in the hospital with no neurological deficits. He was recommended to avoid alcohol and outpatient care for both dengue and alcohol cessation. The patient was also advised to continue with close follow-ups for repeat MRI to monitor the thalamic lesions. However, the patient failed to maintain follow-up.

In this instance, the clinical picture of dengue encephalitis and Wernicke’s encephalopathy was so similar that chronic alcoholism and dengue fever jointly resulted in a difficult diagnostic problem. Arriving at a prompt and simultaneous management of both conditions has contributed to a favorable outcome and underlines the importance of evaluating a broad differential diagnosis in patients with a complex medical history.

A brief case summary is shown in Table 1.

**Table 1:** Case summary.

Sequential events	Interpretation with relevant data
Case presentation (brief history and symptoms)	A 35-year-old chronic binge drinker was admitted with fever (102°F), confusion, and signs of alcohol withdrawal, including tremors and agitation. He last consumed alcohol 2 days before admission.
Clinical examination	Examination revealed a toxic appearance, disorientation, fever, tachycardia, hypotension (80/40 mmHg), and tachypnea (22/min). Neurologically, he had neck rigidity with positive meningeal signs, brisk reflexes, a positive Babinski sign, nystagmus, ophthalmoplegia, and an unsteady gait. Bilateral horizontal gaze palsy was noted, suggesting supranuclear involvement, while vertical movements were relatively preserved. Sensory examination was inconclusive.
Investigations	Blood investigation: Reduced serum vitamin B12 and thiamine levels with normal other metabolic parameters and normal routine investigations MRI brain: Figures 1A–C: Hyperintense lesions in bilateral thalamus Figures 2A,B: Meningeal enhancement suggests meningitis. CSF examination: Moderately elevated cells (50 cells/μL) with moderately elevated protein (60 mg/dL) and normal glucose. CSF PCR for dengue antigen was positive Caine’s criteria were fulfilled, supporting the diagnosis of Wernicke’s encephalopathy
Diagnosis	Overlap between dengue meningoencephalitis with possible Wernicke’s encephalopathy
Treatment	In the early part of management, supportive care for dengue fever consists of intravenous fluids to prevent shock and antipyretics to control fever and continuous monitoring of blood counts. Empirical intravenous thiamine supplementation was provided throughout the hospital stay because of the risk of WE. In addition, the patient started showing signs of alcohol withdrawal and was treated with benzodiazepines to control agitation and prevent withdrawal seizures.
Outcome	The patient’s fever improved, and his neurological status improved as well during his stay in the intensive care unit. After thiamine replacement, as gaze restriction improved, horizontal nystagmus became prominent, particularly in lateral gaze. This transition aligns with the well-documented phenomenon in B1 deficiency, where initial gaze restriction improves, but nystagmus—often of a horizontal nature—becomes evident during recovery. He was discharged after approximately 2 weeks in the hospital with no neurological deficits but was subsequently lost to follow-up.

## Discussion

Chronic alcohol use concomitant with a dengue infection is a situation where dengue encephalopathy and Wernicke’s encephalopathy converge on the same patient due to the complex interplay between an infectious disease, nutritional deficiency, and neurological consequences. Research into these conditions examines their pathophysiology, clinical manifestation, and imaging findings, which are essential for accurate differential diagnosis, and we have been able to gain valuable insight into these important conditions (3).

### Understanding dengue encephalopathy and Wernicke’s encephalopathy

Dengue encephalopathy is an uncommon but serious complication of severe dengue virus infection; severe dengue virus infection (4, 5). Approximately 1–5% of dengue cases develop neurological manifestations, and in severe cases, this figure may increase to 21%. These neurological manifestations of dengue usually indicate very severe dengue disease and are associated with poor patient outcomes (4).

Dengue encephalopathy is a rare but serious complication of severe dengue infection, which occurs when the virus, after affecting the peripheral systems, invades the central nervous system (CNS). This invasion can happen directly through a breach in the blood–brain barrier (BBB), either via infected immune cells or via vascular endothelial infection by the dengue virus (DDV). Once inside the CNS, the virus causes inflammation and edema, particularly in the high vascular areas, such as the thalamus (4). In severe dengue, the immune system’s response produces cytokines such as interleukin 6 (IL-6) and tumor necrosis factor alpha (TNF-*α*), which increase BBB permeability and contribute to vasogenic edema. Hyperintense signals observed on MRI correspond to lesions in the thalamus, which result from a combination of direct viral effects and cytokine-driven inflammation, thereby elucidating the development of thalamic lesions in dengue encephalopathy (4).

Severe dengue can also cause microvascular injury, leading to small vessel ischemia. The brain damage observed in dengue encephalopathy, often involving the thalami, which lack large arteries but have small arteries, is believed to result from this ischemic process (4).

As is well documented with chronic alcohol dependence, Wernicke’s encephalopathy can manifest in those with malnutrition, gastrointestinal surgeries, or other causes of thiamine deficiency. Wernicke’s encephalopathy is frequently underdiagnosed; post-mortem studies indicate that up to 80% of cases go undiagnosed. Fewer than 20% of patients present with the classic triad of symptoms: confusion, ataxia, and ocular abnormalities, so clinicians must be vigilant for this disease (5).

The typical lesions of Wernicke’s encephalopathy are usually well-visualized on MRI, commonly involving the thalami, mammillary bodies, and periaqueductal gray matter. The thalami are often affected in both alcoholic and non-alcoholic cases. Studies have suggested that non-alcoholic Wernicke’s cases may have atypical lesion patterns. Overall, this evidence supports consistent thalamic involvement in Wernicke’s encephalopathy, independent of alcoholics (5).

Thiamine deficiency, particularly prevalent in chronic alcoholism, disrupts energy metabolism, as thiamine is a critical cofactor for carbohydrate metabolism. This results in a deficit in energy production in the brain, especially in ‘high demand’ regions such as the thalamus, resulting in cellular dysfunction and swelling visible on MRI. The thalami and mammillary bodies are sensitive to diabetes because of their high glucose demands. In the absence of thiamine, these regions experience metabolic toxicity, leading to cells being damaged and lactate buildup occurring. Furthermore, thiamine deficiency compromises the blood–brain barrier, exacerbating edema and inflammation. Chronic alcohol consumption further weakens the BBB, making its neurotoxic effects worse. Therefore, early thiamine treatment is essential to prevent long-term cognitive impairment, such as that observed in Korsakoff syndrome, which can develop if Wernicke’s encephalopathy is not treated (5).

### Bilateral thalamic lesions and differential diagnosis

Bilateral damages in the thalamus are rare and may be attributed to numerous factors (6, 7). These include vascular events, such as an infarction of the artery of Percheron, as well as infectious causes, all of which should be considered a part of the differential diagnosis. Notable infectious causes affecting the thalamus include West Nile virus, Japanese B encephalitis, and herpes simplex virus encephalitis, all of which can produce bilateral thalamic lesions. Japanese encephalitis, in particular, appears to have a marked propensity to involve the thalamus and is associated with significant neurological signs. Tuberculosis of the central nervous system can cause bilateral thalamic lesions by forming granulomas or tuberculomas (6). Although dengue fever primarily presents with systemic and hemorrhagic manifestations, neurological presentations are unusual. In rare cases, however, dengue can lead to neurological diseases, specifically encephalitis, which may affect the brain or its structures, including the thalamus. Both thalami are affected in dengue encephalitis, especially in severe cases of dengue illness. The process is believed to be immunological, direct viral, or a consequence of remote organ involvement, such as hemorrhage or thrombosis. Thus, rapid diagnosis and prompt supportive treatment of dengue encephalitis are critical to avoid neurological deterioration (7).

Other conditions that may lead to such lesions include metabolic encephalopathies such as Wernicke’s encephalopathy, neurodegenerative disorders, and neoplastic diseases, such as lymphoma. Patients typically present with complex clinical features such as confusion, memory and/or speech difficulties, and motor dysfunction, since the thalamus plays a key role in relaying sensory-motor information. MRI remains the most helpful neuroimaging modality for diagnosing and differentiating these lesions. Thus, differential diagnosis plays a critical role, as management varies significantly depending on the underlying etiology. Early and accurate disease recognition is very important to achieve the best outcomes (7).

### Early thiamine administration and its mechanism

Thiamine, or vitamin B1, is essential for glucose metabolism and neuronal function, making it crucial in the treatment of Wernicke’s encephalopathy (WE), a condition often caused by thiamine deficiency (8, 9). In WE, the lack of thiamine impairs the activity of enzymes such as pyruvate dehydrogenase, transketolase, and *α*-ketoglutarate dehydrogenase, which are vital in the Krebs cycle and the pentose phosphate pathway (8). This disruption hinders ATP production, leading to energy deficits in the brain, particularly in regions such as the thalamus, mammillary bodies, and cerebellum. The result is the accumulation of toxic byproducts such as lactate, contributing to neuronal damage and the characteristic brain lesions observed in WE. Thiamine administration replenishes these enzyme functions, facilitating glucose metabolism and reducing lactate buildup, thereby protecting neurons from oxidative stress and apoptosis (8). Administration of thiamine not only arrests the process of neurological deterioration but can also help regain some of the lost neurologic function, including reversal of symptoms of confusion, ataxia, and ocular abnormalities when the condition is diagnosed early enough (8). Hence, thiamine administration should be considered a part of the practical measures in any management plan in a part of the world where dengue fever is prevalent in patients showing encephalopathy manifestations (9).

This case affirms the significant difficulty in recognizing the difference between dengue meningoencephalitis and Wernicke’s encephalopathy that commonly presents in patients who have risk factors for both illnesses, for instance, chronic alcoholism and an infectious illness, including dengue fever. As in the present case, both conditions present with clinical, radiological, and biochemical features such as bilateral thalamic hyperintense lesions on MRI, changes in mental status, and focal neurological deficits.

### Limitations

One of the largest challenges this case presented was that, after radiological imaging and cerebrospinal fluid analysis, no clear diagnosis could be made. Despite positive serology for dengue and clinical signs suggesting dengue encephalopathy, the patient’s alcoholism, previous history, and proven thiamine deficiency in this case, Wernicke’s encephalopathy could not be ruled out and was kept as an important alternative diagnosis and a co-association. Neuropsychiatric symptoms are present in both conditions, and the bilateral thalamic lesion on the MRI—most characteristic of both diseases—intensified the diagnostic confusion (10).

One of the study’s limitations was not using follow-up imaging, which might have offered a further understanding of the resolution of the thalamic lesions or their progression after the intervention. Unfortunately, because follow-up imaging was not performed, the prolonged neurological effects and relative contributions of the two encephalopathies could not be assessed. The patient also was not adherent to follow-up, which is also a common problem observed in resource-poor settings where patients are difficult to track and may not be able to afford long-term care for their illness.

### Future directions

The diagnostic overlap between dengue encephalopathy and Wernicke’s encephalopathy in patients with multiple risk factors necessitates further research into the distinguishing features of these conditions. Future studies could explore advanced neuroimaging techniques, such as diffusion-weighted imaging (DWI) or MR spectroscopy, which may help differentiate between viral-induced brain edema and thiamine deficiency-related lesions. Additionally, the development of specific biomarkers—whether immunological, metabolic, or radiological—could aid in making diagnoses that are more precise in cases where the etiology is uncertain (10).

Further research into clinical predictors or scoring systems that account for both infectious and nutritional etiologies may also improve the ability to make early and accurate diagnoses in resource-limited settings. Such tools could be instrumental in guiding treatment decisions, particularly in areas where both alcohol use and dengue are prevalent (11).

Finally, the creation of protocols for early intervention, where both conditions are suspected, may lead to better outcomes. For example, immediate empirical thiamine administration, as done in this case, should be considered in any suspected WE case, given the irreversible nature of untreated Wernicke’s encephalopathy (11).

## Conclusion

This case shows the necessity of considering and identifying Wernicke’s encephalopathy (WE) in patients with dengue fever and chronic alcoholism. The patient had a history of alcohol consumption and was thus at high risk of developing WE, which might be overlooked in dengue-endemic areas. Although dengue encephalopathy and WE share similar clinical and radiologic manifestations, early administration of thiamine proved to be an essential intervention to prevent further irreversible neurological sequelae. This case also stresses that WE should not be excluded from the differential diagnosis of encephalopathy in dengue patients, especially in chronic alcoholics. Despite the diagnostic uncertainty of WE, early thiamine treatment significantly improves the overall functioning of the patient. These challenges in differential diagnosis underscore the need for further studies to improve diagnostic approaches and to develop biomarkers and clinical predictions and scoring systems for early identification and intervention in such complex cases.

## Data Availability

The original contributions presented in the study are included in the article/supplementary material; further inquiries can be directed to the corresponding author.
